# A computational study to identify Sesamol derivatives as NRF2 activator for protection against drug-induced liver injury (DILI)

**DOI:** 10.1007/s11030-023-10686-8

**Published:** 2023-07-01

**Authors:** Ajay Mili, Sumit Birangal, Krishnadas Nandakumar, Richard Lobo

**Affiliations:** 1 Department of Pharmacognosy, Manipal College of Pharmaceutical Sciences, Manipal Academy of Higher Education, Manipal, Karnataka 576104 India; 2 Department of Pharmaceutical Chemistry, Manipal College of Pharmaceutical Sciences, Manipal Academy of Higher Education, Manipal, Karnataka 576104 India; 3 Department of Pharmacology, Manipal College of Pharmaceutical Sciences, Manipal Academy of Higher Education, Manipal, Karnataka 576104 India

**Keywords:** NRF2, Drug-induced liver injury (DILI), Sesamol derivatives, Oxidative stress, Hepatoprotective, Computational techniques

## Abstract

Drug-induced liver injury can be caused by any drugs, their metabolites, or natural products due to the inefficient functioning of drug-metabolizing enzymes, resulting in reactive oxygen species generation and leading to oxidative stress-induced cell death. For protection against oxidative stress, our cell has various defense mechanisms. One of the mechanisms is NRF2 pathway, when activated, protects the cell against oxidative stress. Natural antioxidants such as Sesamol have reported pharmacological activity (hepatoprotective & cardioprotective) and signaling pathways (NRF2 & CREM) altering potential. A Computational analysis was done using molecular docking, IFD, ADMET, MM-GBSA, and Molecular dynamic simulation of the Schrödinger suite. A total of 63,345 Sesamol derivatives were downloaded for the PubChem database. The protein structure of KEAP1-NRF2 (PDB: 4L7D) was downloaded from the RCSB protein database. The molecular docking technique was used to screen compounds that can form an interaction similar to the co-crystalized ligand (1VX). Based on MM-GBSA, docking score, and interactions, ten compounds were selected for ADMET profiling and IFD. After IFD, five compounds (66867225, 46148111, 12444939, 123892179, & 94817569) were selected for molecular dynamics simulation (MDS). Protein–ligand complex stability was assessed during MDS. The selected compounds (66867225, 46148111, 12444939, 123892179, & 94817569) complex with KEAP1 protein shows good stability and bond retentions. In our study, we observed that the selected compounds show good interaction, PCA, *R*_g_, binding free energy, and ADMET profile. We can conclude that the selected compounds can act as NRF2 activators, which should be validated using proper in-vivo/in-vitro models.

**Graphical abstract:**

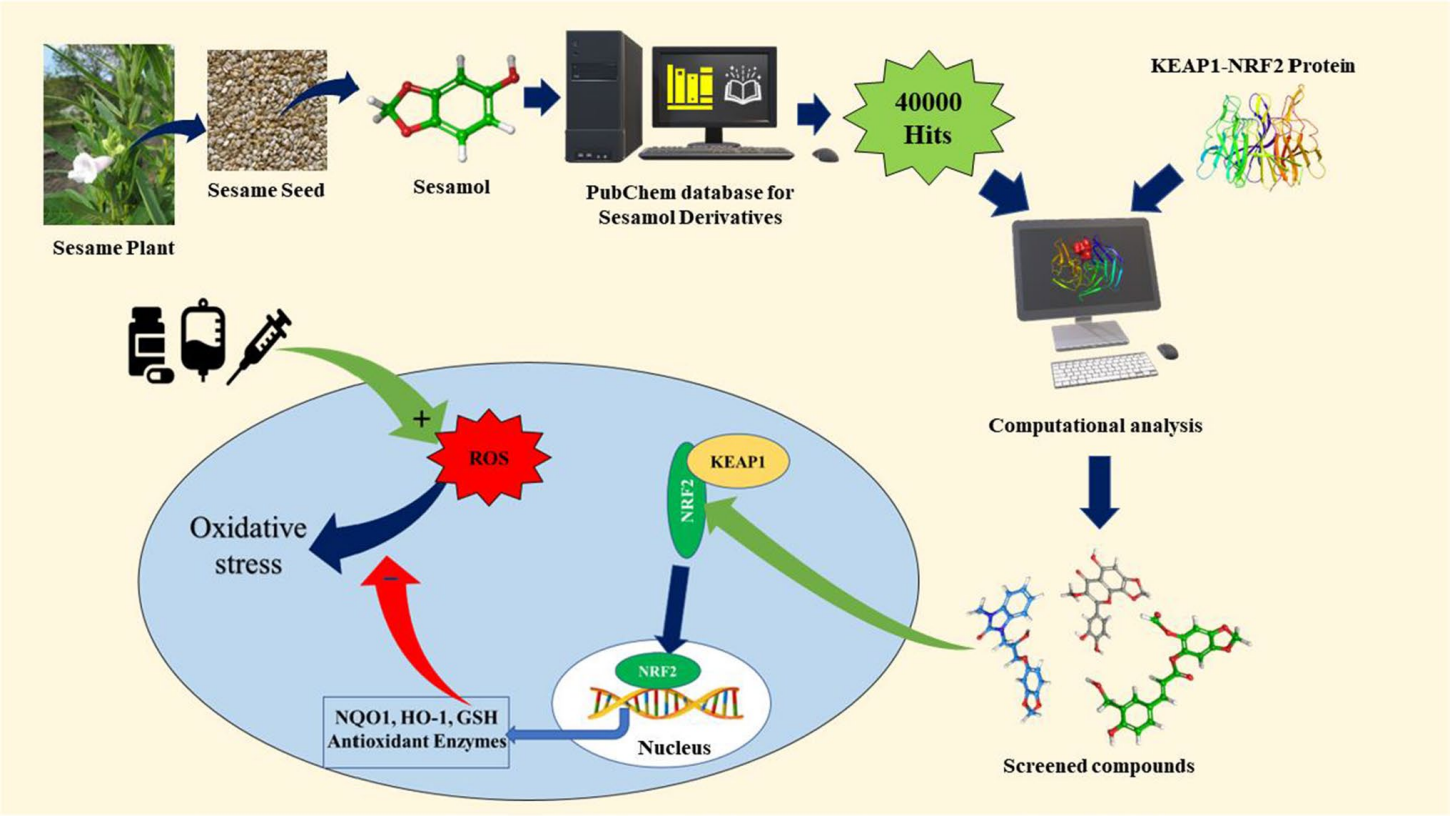

## Introduction

Drug-induced liver injury (DILI) is defined “as a liver injury caused by various medications, herbs, or other xenobiotics, leading to abnormalities in liver tests or liver dysfunction with the reasonable exclusion of other etiologies” [1]. Based on the damage site and injury period, it is categorized as hepatitis, cholestatic, or a mixed pattern of injury and chronic or acute [2]. According to reports, the DILI incidence rate is 1.3–19.1 per 100,000 people worldwide [3–5]. The exact incidence rate of DILI is complicated to estimate as most cases are underreported and/or due to the utilization of a wide range of diagnostic criteria [6, 7]. It can be triggered by a variety of drugs, including antituberculosis, chemotherapy drugs, and antipyretics analgesics. DILI-related problems are one of the major factors in the design of new drugs as well as restriction, withdrawal, or project termination of drug candidates and existing drugs. The complexity underlying the pathophysiology of DILI has gathered interest from the scientific community in recent years. During the 1950s, scientists uncovered a connection between drug-reactive metabolites and DILI, and it has been an area of interest ever since. Several studies have associated mitochondrial dysfunction, inflammatory responses, and oxidative stress with the occurrence and development of DILI [3–5, 8]. DILI could be linked to direct toxicity from the prescribed drug or its metabolites, or it may be caused by immune-mediated processes. Even though the mechanisms are different, it is interlinked, e.g., drug-induced toxicity leads to hepatocyte damage, which may be exacerbated by successive inflammatory reactions [2].

Most drugs are metabolized inside the liver and then excreted in bile or urine. Drug metabolism involves two reactions, phase-I and phase-II, which have their own enzymes that work in unison for the metabolism and excretion of the drug from our body. The initial step in drug metabolism involves the phase-I enzymes, which react with the drug to form an intermediate product. The intermediate product formed during the phase-I reaction was inactivated in the phase-II reaction and excreted out. Any difference in the working of enzymes during phase-II leads to the accumulation of toxic metabolites. These toxic metabolites may interact with numerous cellular organelles (e.g., mitochondria), resulting in the generation of reactive oxygen species (ROS), which intensifies oxidative stress, which can directly damage enzymes, protein, and DNA inside cells and tissues and also prompt immune-mediated liver injury [9, 10].

Nuclear factor erythroid 2-related factor 2 (NRF2) is a member of the cap'n'collar basic leucine zipper family located in the cytoplasm of the cell [11, 12]. The NRF2 has two motifs, i.e., Aspartic acid–Leucine–Glycine (DLG) and Glutamic acid–Threonine–Glycine–Glutamic acid (ETGE), which bound it with Kelch-like ECH-associated protein 1 (KEAP1) protein. DLG motif of NRF2 binds with SER (363, 508, 555 & 602), ARG (380, 415 & 483), ASN 382, GLN 530, and TYR 525, and ETGE motif binds with SER (363, 508, 555 & 602), ARG (380, 415 & 483), ASN 382, GLN 530, and TYR 525 amino acid residue of KEAP1 protein, respectively [13]. Under normal conditions, NRF2 remains bounded with a KEAP1, and this bounded NRF2 is ubiquitination and degraded by the proteasome. Under stress conditions, the bonding between the KEAP1 and the ETGE and DLG motif of NRF2 disrupts. The free NRF2 translocates inside the nucleus, where it gets bound with the antioxidant response element. Initiating the transcription of the genes, including glutathione-S-transferases and heme-oxygenase-1, for the production of phase-II enzymes and antioxidant proteins, decreases the sensitivity toward oxidative stress [14, 15]. Recently, research has been carried out to identify substances that can destabilize the protein–protein interaction of KEAP1-NRF2, freeing NRF2 for protection against oxidative stress [13, 16]. The activation of NRF2 has the potential to provide protection against liver damage caused by DILI resulting from oxidative stress, which makes it a potential target for further research.

Natural antioxidants have always been an area of interest for researchers due to their potential to attenuate various disease conditions like cardiovascular diseases [17, 18], gastric ulcers [19, 20], Cancer [21, 22], Diabetes [23, 24], hepatotoxicity [25, 26], and inflammation [8, 9]. Sesamol is a natural antioxidant isolated from the *Sesamum indicum* L. plant seed oil. Sesamol has a variety of biological activities including lipid peroxidation inhibition and ROS scavenging [27], antioxidant enzyme upregulation [28], hepatoprotective [29], cardioprotective [30], and neuroprotective [31] activities. Most of its activity is linked to its antioxidant activity and signaling-pathway (like NRF2 & CREM) altering potential [32–34]. However, no studies are available to identify Sesamol derivatives as NRF2 activators. Hence, computational studies such as molecular docking, MM-GBSA, Induced fit docking, ADMET, and Molecular dynamic simulation were employed to identify Sesamol derivatives that can activate the NRF2 pathway and protect against DILI via mediating oxidative stress.

## Material and method

I*n silico* studies including ligand docking, MM-GBSA, induced fit docking, ADMET, and molecular dynamics were performed using Glide module “Ligand docking,” Prime module “MM-GBSA,” “Induced fit docking,” “QikProp,” and “Desmond” of Schrödinger suite Version 11.4. Principal component analysis (PCA) was done using Visual molecular dynamic (VMD).

### Protein preparation and grid generation

KEAP1-NRF2 crystal structure (PDB id: 4L7D) with human origin was downloaded from the RCSB protein data bank (https://www.rcsb.org/structure/4L7D). The selected PDB has a Resolution of 2.25 Å, RMSD value of 0.8682 Å, and IC_50_ of the co-crystalized Ligand (1VX) was 0.75 ± 0.32 μmol. 1VX is an Nrf2 activator and a direct inhibitor of the Nrf2-Keap1 complex formation. It was identified through a high-throughput screen of small molecules and has shown promising binding interactions with the Keap1 protein [16].

Before beginning the molecular simulation studies, the Schrödinger suite module “Protein Preparation Wizard” was used to prepare protein. During the protein preparation process, the protein was imported, refined, reviewed, modified, and minimized. Side chains and missing residue were filled using the prime tool. Catalytically significant residue and active sites were preserved in the protein structure. Beyond 5 Å, water molecules were removed, and stage for heteroatoms was generated. OPLS3e (Optimized potential for liquid stimulation) force field was used for energy minimization and generating low-energy state protein. Molecular modeling was done using this protein. The panel “Receptor grid generation” was used for generating a grid encompassing the ligand at coordinate *X* (− 22.79), *Y* (39.11), & *Z* (– 36.57) while keeping all the functional residue inside the grid in default [35–37].

### Ligand preparation

The structures of around 63,345 compounds with Sesamol as a basic ring were downloaded from the PubChem database (https://pubchem.ncbi.nlm.nih.gov/#query=CID68289%20structure&tab=substructure&fullsearch=true&page=1). After Lipinski’s Rule of 5 screening, out of 63,345 downloaded ligand only 41,786 ligand remained. The LigPrep tool was used for ligand preparation. Using the OPLS3e force field, structures with the lowest energy and co-related chirality were prepared at pH 7.4 ± 0.0. The ligand was preprocessed including tautomers generation, ionization at pH 7.4 ± 0.0 by Epik, H-bond addition, neutralization of charged group, and optimization of ligand geometry [35–37].

### Ligand docking

Ligand docking studies were performed using the GLIDE (Grid-based Ligand Docking with Energetics) module. High-throughput Virtual Screening (HTVS), Standard precision (SP), and Extra precision (XP) were used to get different scoring functions. Firstly, HTVS docking was used to dock all the ligands. As HTVS docking computationally lacks explicit water technology and descriptor, the top 5 percent of the ligands were again analyzed using SP and XP modes to avoid false-positive results [35–37].

### Free ligand binding energy (MM-GBSA) calculation

MM-GBSA method was used to find the absolute Ligand binding affinities of the top 100 ligands. It facilitates in comprehending the binding of protein–ligand which will persist long enough to elicit biological response [38].

### ADMET

After XP docking and MM-GBSA, the top ten ligands were selected, and ADME analysis was done using the QikProp tool. QPlogS (Predicted aqueous solubility), QPlogHERG (cardiotoxicity), QPlogPo/w (predicted octanol/water coefficient), QPPCaco (Cell permeability), QPlogHERG (cardiotoxicity), QPlogBB (Predicted brain/blood partition coefficient), and Lipinski's rule of five were established using this process. The toxicity analysis studies was done using “admetSAR” [39].

### Induced fit docking (IFD)

IFD of the top 10 selected compounds were performed where 20 was set as the maximum pose for each ligand, and 0.50 was kept as the van der Waals scaling. After that, prime side chain prediction and energy minimization were conducted, and all residue refinement was done for side chains and ligand poses. The induced fit protein structure underwent extensive XP docking, and the IFD dock score was computed [40–42].

### Molecular dynamic simulations (MDS)

For investigating the dynamics and function of protein–ligand complexes, MDS is used widely. Ligand docking does not replicate the biological environment where the ligand and protein are dissolved in water. Therefore, MDS was applied to resolve the issue. Depending on the ligand docking score, poses, MM-GBSA, ADMET profile, and IFD data, compounds will be selected for MDS for 100 ns. Before beginning the MDS, the complete system was submerged inside a simple point charge (SPC) solvent model. Throughout the system-building process, the boundary condition's orthorhombic box form was maintained. The Buffer box size calculation method is utilized throughout the building process, and the distance (Å) was *a* = 10, *b* = 10 & *c* = 10, and the angle was *α* = 90֯, *β* = 90֯, *γ* = 90֯, respectively. The System Builder tool was used to prepare the system for MD simulation, and the OPLS3e force field was employed throughout the system preparation process. Minimization tool was used for system minimization. Finally, a simulation time of 100 ns was selected, where every 100 picoseconds 1 frame was captured from the trajectory, and 1000 frames were generated throughout the MDS process. NPT (constant particle number (N), pressure(P) 1.01325 bar, and temperature (T) 300 K) ensemble were used for the production run with OPLS3e as the chosen force field for the MDS process. Lastly, the Simulation interaction diagram tool was used to generate the report of the MDS [40–42].

## Result and discussion

PDB (4L7D) for KEAP1-NRF2 from human origin was downloaded from the “rscb.org” portal. The selected PDB has an RMSD value of 0.8682. 63,345 Ligands were downloaded from the PubChem database. Protein and ligand were prepared at 7.4 ± 0.0 pH. All the procedures are followed as mentioned in the material and method.

### Molecular docking and MM-GBSA

Prepared ligands were docked using HTVS docking mode, the ligands having a score more than − 6 kcal/mole were selected and again analyzed in SP docking mode. Again, the top five percent of ligands were subjected to the XP docking mode. XP docking mode provides better accuracy, decreases false-positive results, and provides an improved association between ligand pose and docking score. Finally, 100 compounds with pivotal interaction and a good XP dock score were selected. Ligand structure, MM-GBSA score, and docking score of the top 10 compounds and 1VX (co-crystalized ligand) are shown in Table 1, and 2d interactions are shown in Table 2.

**Table 1:** Ligand structure, Docking score, and MM-GBSA of the co-crystal ligand and top 10 selected compound

Title	Structure	MM-GBSA (kcal/mol)	Docking score (kcal/mol)
IVX		− 86.39	− 7.038
147327805		− 102.22	− 12.191
24761003		− 94.90	− 11.541
149283896		− 83.96	− 11.084
101538092		− 63.35	− 10.495
66867225		− 97.53	− 9.077
46148111		− 83.96	− 8.963
12444,939		− 65.51	− 8.883
123892179		− 64.13	− 8.624
94817569		− 73.46	− 7.09
119729222		− 76.91	− 7.526

**Table 2:** Interaction diagram and Interaction of 1VX (co-crystalized ligand) and top 10 ligands

Title	2D interaction diagram	Interactions
IVX		H-bond: SER602, ARG415, ASN414 H-bonded water bridge: ASN382, SER363 Salt bridge: ARG380 pi–pi stacking: TYR572
147327805		H-bond: VAL606, VAL604, ILE559, GLY367 H-bonded water bridge: ARG415 π-cation stacking: ARG415
24761003		H-bond: VAL606, VAL604, VAL463 GLY367 H-bonded water bridge: SER363 π-cation stacking: ARG415
149283896		H-bond: VAL606, VAL604, SER602, ILE559, ARG415 H-bonded water bridge: SER363 pi–pi stacking: TYR334 π-cation stacking: ARG415
101538092		H-bond: SER602, ILE416, LEU365 π-cation stacking: ARG415
66867225		H-bond: VAL604, SER602, TYR572 pi–pi stacking: TYR334 π-cation stacking: ARG415
46148111		H-bond: SER602, ASN414 H-bonded water bridge: SER363 pi–pi stacking: TYR572, TYR334 π-cation stacking: ARG415
12444939		H-bond: ILE416, LEU365 H-bonded water bridge: SER508, ASN382, SER363 π-cation stacking: ARG415
123892179		H-bond: VAL463, ILE416, ARG415, ASN414 H-bonded water bridge: ASN382, SER363 pi–pi stacking: TYR572
94817569		H-bond: ARG415, ASN382 H-bonded water bridge: SER363 π-cation stacking: TYR334
119729222		H-bond: SER602, ILE416, LEU365 H-bonded water bridge: ASN382, SER363 π-cation stacking: TYR572, TYR334

*H-bond* hydrogen bond, *ARG* arginine, *ASN* asparagine, *ILE* isoleucine, *LEU* leucine, *SER* serine, *TYR* tyrosine, *VAL* valine

The 1VX (co-crystalized ligand) forms H-bond with SER602, ARG415 & ASN414; pi–pi stacking with TYR572; H-bonded water bridge with SER363; and salt bridge with ARG380. The compound 147327805 forms H-bond with VAL (606 & 604), ILE559, and GLY367; *π*-cation stacking and an H-bonded water bridge with ARG415. The compound 24761003 forms H-bond with GLY367, VAL (606, 604, & 463); π-cation stacking with ARG415; and an H-bonded water bridge with SER363. The compound 149283896 forms H-bond with VAL (606 & 604), SER602, ILE559, and ARG415; an H-bonded water bridge with SER363; *π*-cation stacking with ARG415; and pi–pi stacking with TYR334. The compound 101538092 forms a *π*-cation stacking with ARG415, and H-bond with SER602, ILE416, and LEU365. The compound 66867225 forms a *π*-cation stacking with ARG415, a pi–pi stacking with TYR334, and an H-bond interaction with VAL604, SER602, and TYR572. The compound 46148111 forms a *π*-cation stacking with ARG415, pi–pi stacking with TYR (572 & 334), and H-bond with SER602 and ASN414. The compound 12444939 forms a π-cation stacking with ARG415, an H-bonded water bridge with SER (508 & 363) and ASN382, and H-bond with ILE416 and LEU365. The compound 123892179 forms a pi–pi stacking with TYR572, an H-bonded water bridge with ASN382 and SER363, and an H-bond with VAL463, ILE416, ARG415, and ASN414. The compound 94817569 forms a π-cation stacking with TYR334, H-bond with ARG415 and ASN382, and an H-bonded water bridge with SER363. The compound 119729222 forms a *π*-cation stacking with TYR (572 & 334), H-bonded water bridge with ASN382 and SER363, and H-bond with SER602, ILE416, and LEU365 (Table 2). This study found that most compounds show interactions with amino acids ASN382, SER (363 & 602), ARG415, and TYR572 resembling 1VX.

The selected ligands, protein–ligand complex binding free energy, were calculated using the Prime MM-GBSA module. The Binding free energy of the 1VX (co-crystalized ligand) was − 86.39 kcal/mol, and the top 10 compounds ranges from − 102.22 to − 63.35 kcal/mol, shown in Table 1.

### ADMET analysis

After the ligand XP docking and MM-GBSA, ADMET properties of the selected ligands and 1VX were calculated using the QikProp tool. In the drug-designing process, drug-like properties and ADME properties are key filtration criteria, and they are presented in Tables 3 and 4. All the top 10 compounds show good percent oral absorption, with compounds 46148111 and 94817569 showing 100% absorption. Other properties like H-bond donor (range: 0.0–6.0), acceptor (range: 2.0–20.0), and QPlogPo/w (range: − 2.0 to 6.5) were all within the range. Also, all the compounds follow the Lipinski's rule of five.

**Table 3:** ADMET profile of selected compounds

Title	Molecular weight (g/mol)	H-bond donor	H-bond acceptor	#metab	PSA	% Human oral absorption	Rule of five
147327805	462.449	3	10.15	3	123.855	89.071	0
24761003	398.368	4	8.9	5	140.084	65.739	0
149283896	494.493	3	9.4	2	116.4	91.815	0
101538092	330.25	3	6	4	134.011	64.612	0
66867225	449.508	2	7.25	2	110.935	90.347	0
46148111	464.477	2	9.2	4	124	100	0
12444939	344.277	2	6	4	121	76.641	0
123892179	358.304	2	8.95	2	142.1	69.014	0
94817569	342.351	1	5.95	2	80.009	100	0
119729222	368.432	3	5.75	5	83.284	89.492	0
IVX	446.5	1	8	7	NA	86.623	0

*PSA* van der Waals surface area of polar nitrogen and oxygen atoms and carbonyl carbon atoms, *#metab* number of likely metabolic reactions

**Table 4:** ADMET properties of selected compounds

Title	QPlogKhsa	QPlogBB	QPlog hERG	QPlogS	QPP Caco	QPlog Po/w
147327805	− 0.275	− 1.359	− 6.146	− 4.36	434.468	2.547
24761003	− 0.38	− 2.222	− 5.346	− 3.353	66.391	1.056
149283896	0.046	− 1.324	− 6.374	− 4.893	359.756	3.266
101538092	− 0.284	− 1.614	− 4.425	− 2.779	72.3	0.75
66867225	0.599	− 0.638	− 6.496	− 4.934	219.103	3.673
46148111	0.204	− 1.123	− 6.203	− 5.504	757.906	3.383
12444939	− 0.162	− 1.22	− 4.408	− 3.081	200.247	1.452
123892179	− 0.539	− 1.921	− 5.425	− 2.932	110.729	0.936
94817569	0.027	− 0.444	− 5.36	− 3.766	2154.497	3.032
119729222	0.22	− 0.193	− 5.877	− 3.118	387.529	2.77
IVX	0.008	− 0.675	− 1.224	− 4.095	142.838	3.605

*QPlogKhsa* prediction of binding to human serum albumin, *QPlogBB* predicted brain/blood partition coefficient, *QPlog hERG* predicted IC_50_ value for blockage of HERG K^+^ channels, *QPlogS* predicted aqueous solubility, *QPPCaco* predicted apparent Caco-2 cell permeability in nm/sec, *QPlog Po/w* predicted octanol/water partition coefficient

Pharmacokinetic parameters, mainly QPlogKhsa, QPlogBB, QPlogHERG, and QPPCaco were accessed and are shown in Table 4. The QPlogKhsa values of the selected compounds were in the range of − 0.539 to 0.599, within the range or recommended value of − 1.5 to 1.5. The QPlogBB normal range is − 3.0 to 1.2, and all the compounds were in the range of − 2.222 to − 0.193. The QPlogHERG of all the compounds was in the range of − 6.374 to − 4.408. The QPPCaco-2 permeability of all the compounds ranged from 72.3 to 2154.497, with compounds 46148111 and 94817569 showing great permeability, i.e., 757.906 and 2154.497, respectively. Toxicity predicted by “admetSAR” is shown in Table 5.

**Table 5:** Toxicity properties of selected compounds

Compound	Acute oral toxicity	Blood–brain barrier	Carcinogenicity	Hepatotoxicity	Human ether-a-go-go-related gene inhibition	Mitochondrial toxicity	Nephrotoxicity
1VX	III	+	−	−	−	+	−
147327805	III	−	−	+	+	+	+
24761003	III	−	−	+	−	+	+
149283896	III	−	−	+	+	+	−
101538092	III	−	−	+	–	+	−
66867225	III	+	−	−	**+**	+	−
46148111	III	+	−	−	**+**	+	−
12444939	III	–	−	−	–	+	−
123892179	III	–	−	−	–	+	−
94817569	III	+	−	−	–	+	−
119729222	III	+	−	−	+	+	+

− low risk, + high risk and III medium risk

### Induced fit docking (IFD)

**Fig. 1:**
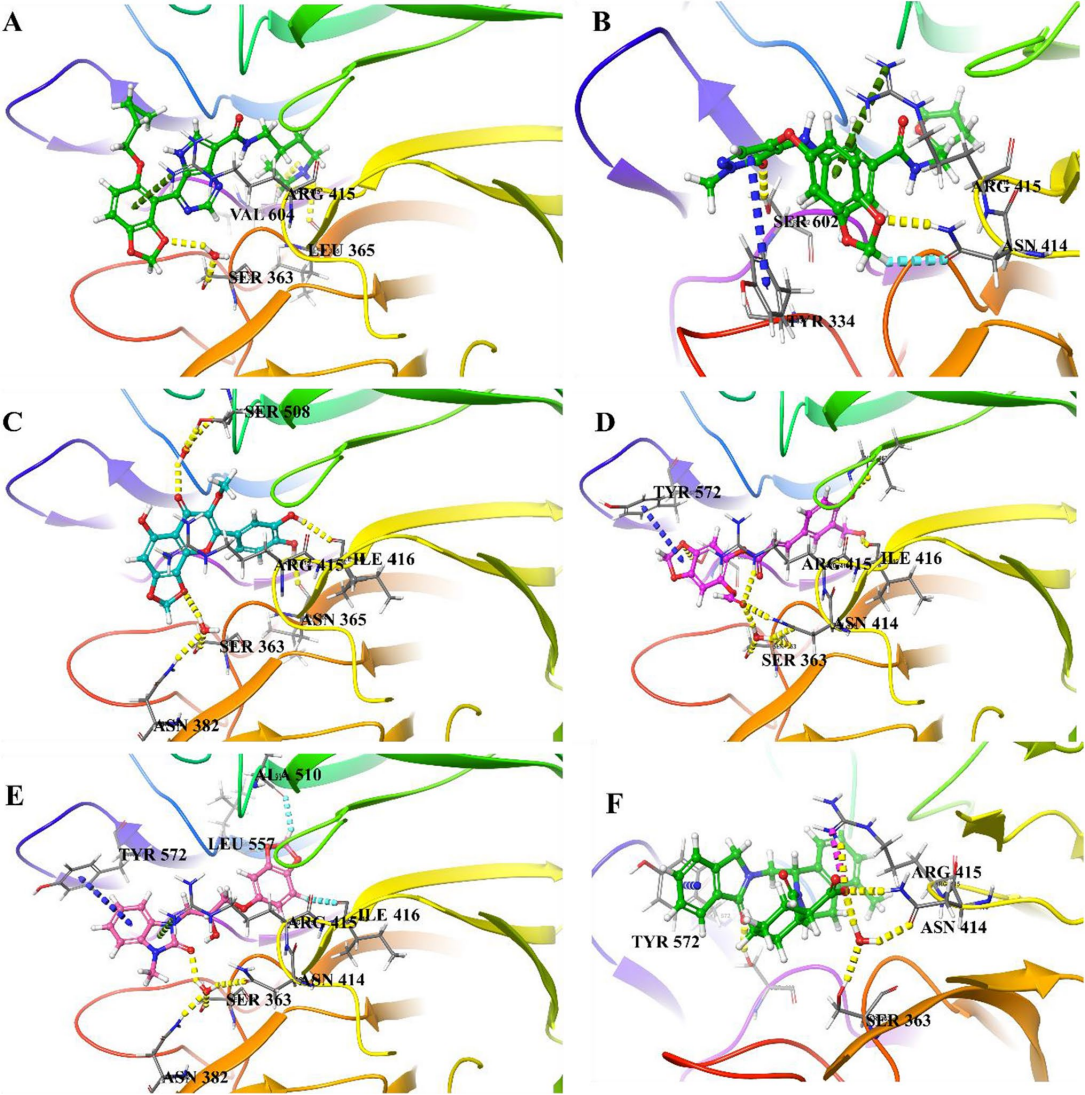
IFD 3D interaction diagram—**a** 66867225 (IFD score: − 619), **b** 46148111 (IFD score: − 616.58), **c** 12444939 (IFD score: − 622.94), **d** 123892179 (− 617.93), **e** 94817569 (IFD score: − 613.89), and **f** 1VX (IFD score: − 641.95)

Ten compounds were chosen for IFD analysis depending on their interactions, XP docking score, and ADMET. After IFD, five compounds, namely, 66867225, 46148111, 12444939, 123892179, and 94817569, were selected for Molecular dynamic analysis by virtue of their IFD score and bond retention. The interaction image and IFD score of the co-crystallized ligand and selected five compounds are shown in Fig. 1.

### Molecular dynamics simulation (MDS)

The main objective of Molecular dynamic simulation is to subject the receptor–ligand complex to the physiological condition that was not possible during ligand docking or IFD. Depending on the docking score, ADMET profile, MM-GBSA, and IFD score, five compounds, namely, 66867225, 46148111, 12444939, 123892179, and 94817569, were selected for MDS analysis. The complex of KEAP1 with selected compounds, Complex-1 (KEAP1-66867225), Complex-2 (KEAP1-46148111), Complex-3 (KEAP1-12444939), Complex-4 (KEAP1-123892179), Complex-5 (KEAP1-94817569), and Complex-6 (1VX-KEAP1) was formed for executing the MDS. “Root means square fluctuation (RMSF),” “Root mean square deviation (RMSD),” and “Lig fit prot” analysis was also performed.

#### Protein–ligand complex root mean square deviation (RMSD) and Lig fit prot

**Fig. 2:**
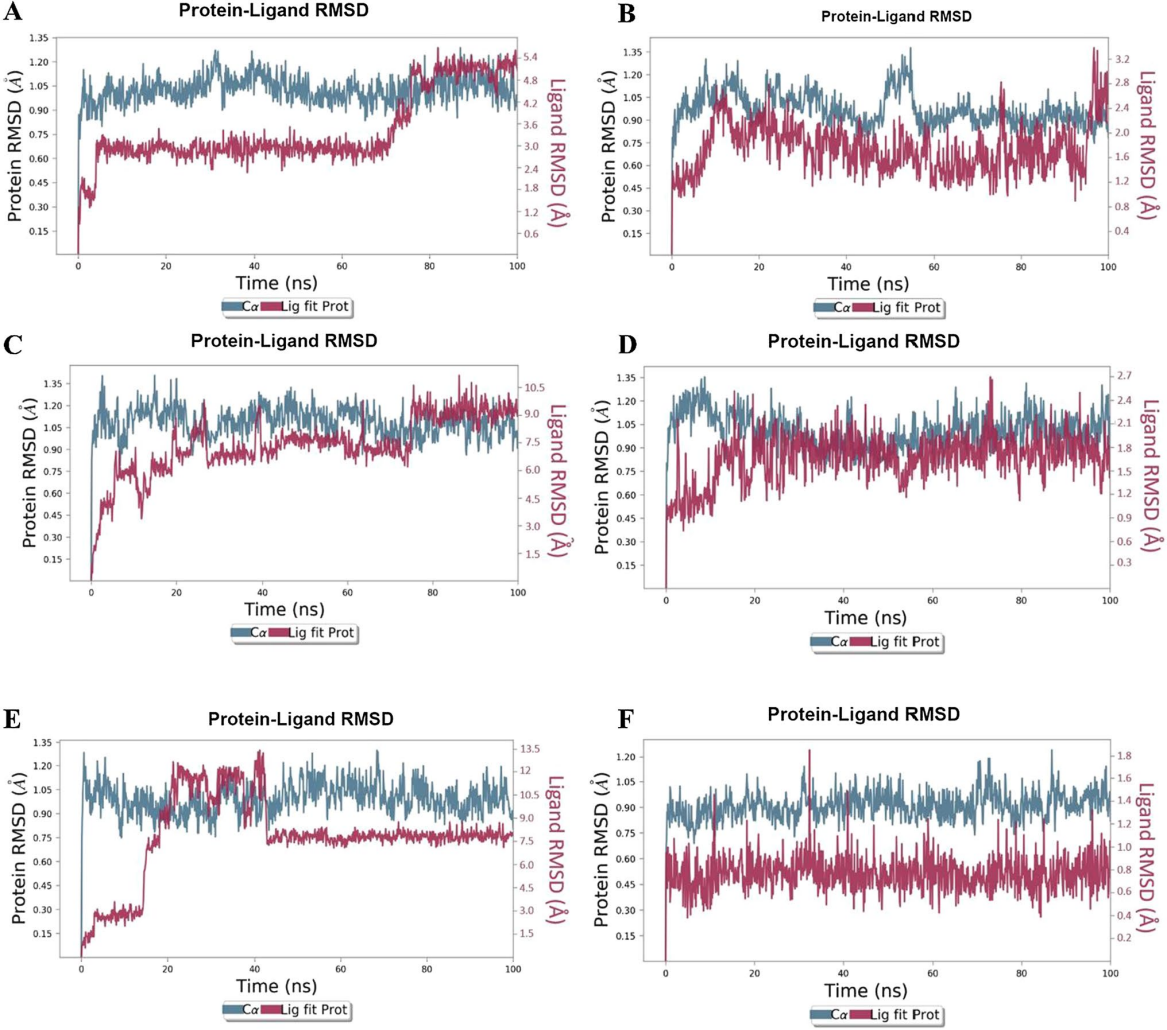
RMSD plot of **a** Complex-1, **b** Complex-2, **c** Complex-3, **d** Complex-4, **e** Complex-5, and **f** Complex-6

The Lig fit prot and RMSD of protein and protein–ligand complex were computed by aligning the generated frame with the reference frame. Complex-1 was stable in the beginning with a slight drift at 4 ns and became stable till 70–75 ns, where a slight drift is observed, after which it stabilizes toward the end (Fig. 2a). In Complex-2, there is a slight drift observed at 6.5–14 ns, after 14 ns, the complex becomes stable till 95 ns, with a slight drift observed at 95.3 ns, and it becomes stable till the end (Fig. 2b). In Complex-3, a drift is observed during the initial stage of simulation at 4.8 ns, 10.4 ns, 18.4 ns, & 26.4 ns after that, it becomes stable, and again a slight drift is observed at 73.6 ns, and it became stable toward the end of the simulation (Fig. 2c). In Complex-4, some minor deviation was observed initially, and it became stable throughout the simulation (Fig. 2d). In Complex-5, a deviation was observed at 15 ns & 42 ns, which stabilizes after 43 ns till 100 ns (Fig. 2e). The Complex-6 was stable throughout the entire simulation (Fig. 2f).

#### Root mean square fluctuation (RMSF)

**Fig. 3:**
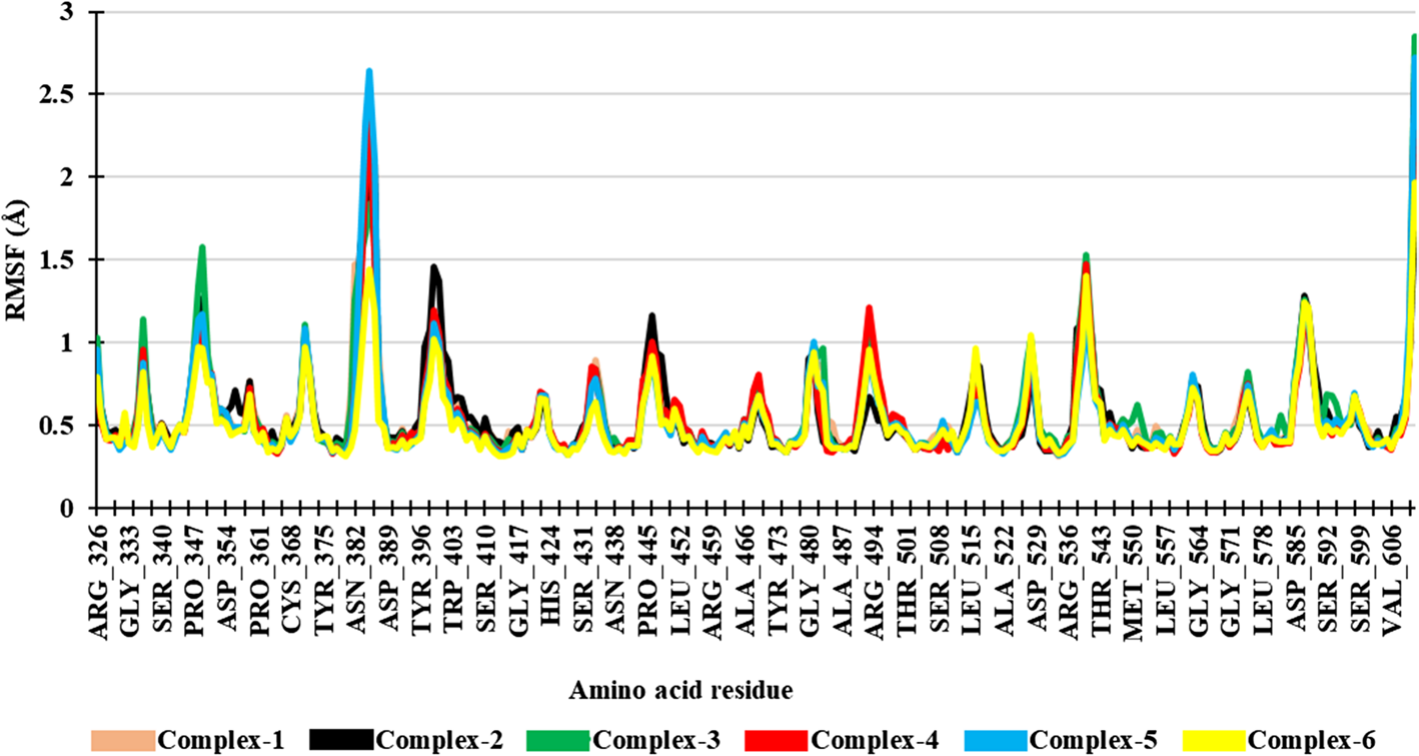
RMSF plot of Complexes

RMSF identifies the residues that contribute to fluctuations in the structure of the complexes and demonstrates local alterations along the protein chain. The RMSF values of key amino acid residues, namely, SER363, ARG380, ASN382, ARG415, ARG483, TYR525, GLN530, SER508, SER555, and SER602, were determined (Fig. 3).

In Complex-1, the RMSF values of the key amino acid residues are 0.388 Å, 0.537 Å, 1.477 Å, 0.463 Å, 0.87 Å, 0.48 Å, 0.472 Å, 0.461 Å, 0.498 Å, and 0.395 Å, respectively. In the case of Complex-2, the RMSF values are 0.423 Å, 0.387 Å, 0.864 Å, 0.376 Å, 0.403 Å, 0.416 Å, 0.396 Å, 0.428 Å, 0.376 Å, and 0.388 Å, respectively. For Complex-3, the key amino acid residue RMSF values are 0.378 Å, 0.373 Å, 1.259 Å, 0.421 Å, 0.971 Å, 0.501 Å, 0.48 Å, 0.417 Å, 0.451 Å, and 0.391 Å, respectively. In Complex-4, the key amino acid residue RMSF values are 0.399 Å, 0.332 Å, 0.736 Å, 0.347 Å, 0.464 Å, 0.424 Å, 0.444 Å, 0.35 Å, 0.38 Å, and 0.394 Å, respectively. In Complex-5, the RMSF values of the key amino acid residues are 0.366 Å, 0.34 Å, 0.714 Å, 0.367 Å, 0.763 Å, 0.473 Å, 0.423 Å, 0.442 Å, 0.428 Å, and 0.375 Å, respectively. For Complex-6 (co-crystallized ligand), the RMSF values of key amino acid residues are 0.339 Å, 0.317 Å, 0.475 Å, 0.323 Å, 0.702 Å, 0.452 Å, 0.452 Å, 0.432 Å, 0.395 Å, and 0.389 Å, respectively. Throughout the entire simulation, the fluctuation observed in the key amino acid residues of the complexes remained stable, indicating minimal conformational changes after the binding of ligands in the pocket. The observed fluctuation was similar to that of the co-crystallized ligand (Complex-6) Fig. 6.

#### Protein–ligand complex interaction

**Fig. 4:**
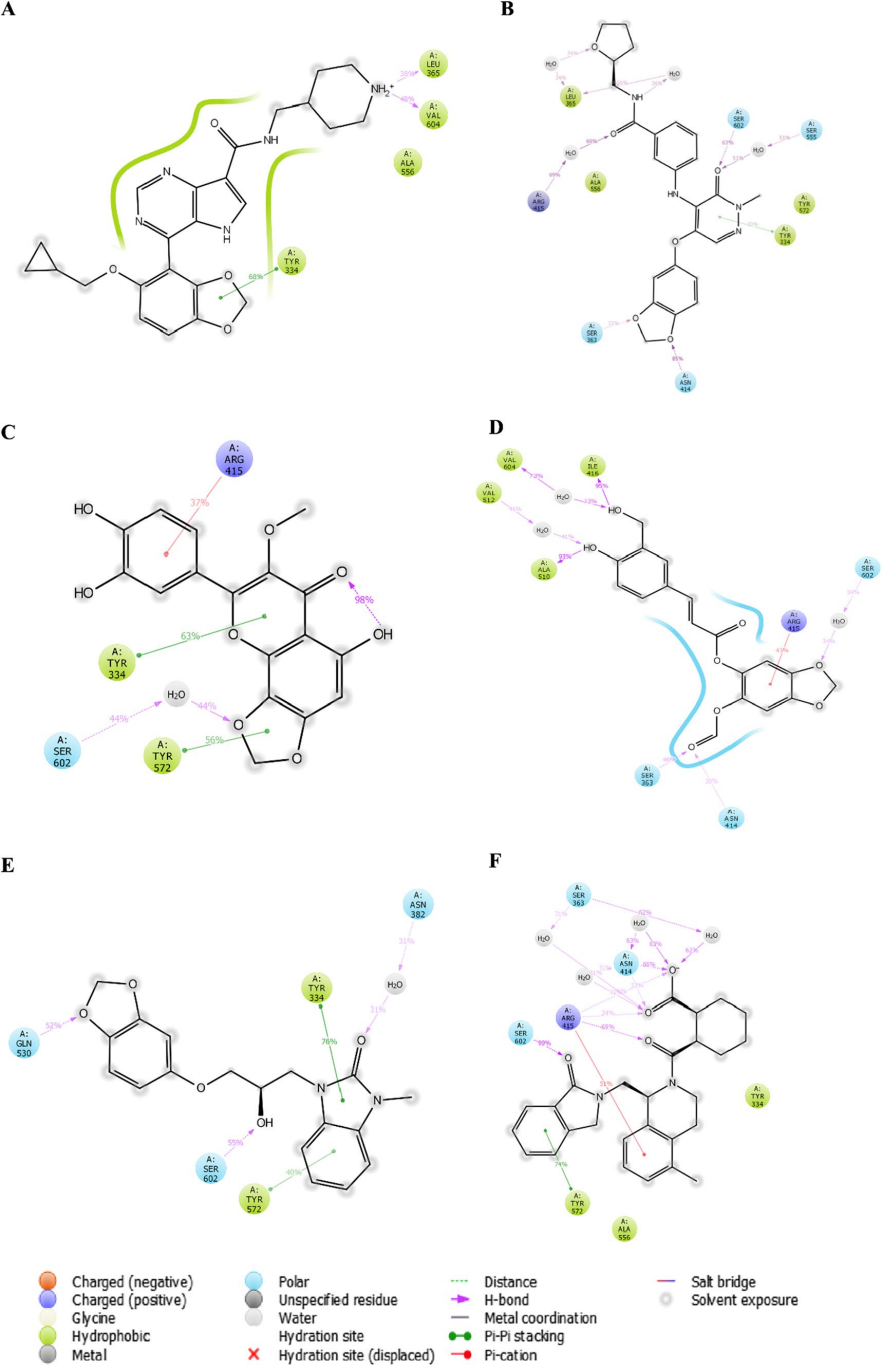
Interaction diagram of—**a** Complex-1, **b** Complex-2, **c** Complex-3, **d** Complex-4, **e** Complex-5, and **f** Complex-6

The interaction of complex-1 with KEAP1 protein shows that it forms hydrogen-bond interaction with VAL604, ALA556, LEU365, and TYR334 (Fig. 4a). In complex-2, it forms H-bonded water bridge with SER555, ARG415, and LEU365; H-bond with SER (363 & 602) & ASN414; and pi–pi stacking with TYR334 (Fig. 4b). In complex-3, TYR (334 & 572) forms pi–pi stacking, π-cation with ARG415, and H-bonded water bridge with SER602, respectively (Fig. 4c). In complex-4, ALA510, ILE416, ARG415, ASN414, and SER363 form H-bond interaction and an H-bonded water bridge with VAL (512 & 604) & SER602 (Fig. 4d). In complex-5, an H-bonded water bridge with ASN382, pi–pi stacking with TYR (334 & 572), H-bond with GLN530 and SER602 (Fig. 4e). In complex-6, ASN414, ARG415, and SER602 forms H-bond, an H-bonded water bridge with ASN414, ARG (380 & 415), and SER363; π-cation and pi–pi stacking with ARG415 and TYR572, respectively (Fig. 4f). It was found that most of the selected compounds maintain interactions with the key amino acids; TYR (334 & 572), SER (363 & 602), ASN414, and ARG415, similar to 1VX (co-crystalized ligand).

#### Protein–ligand contact timeline

**Fig. 5:**
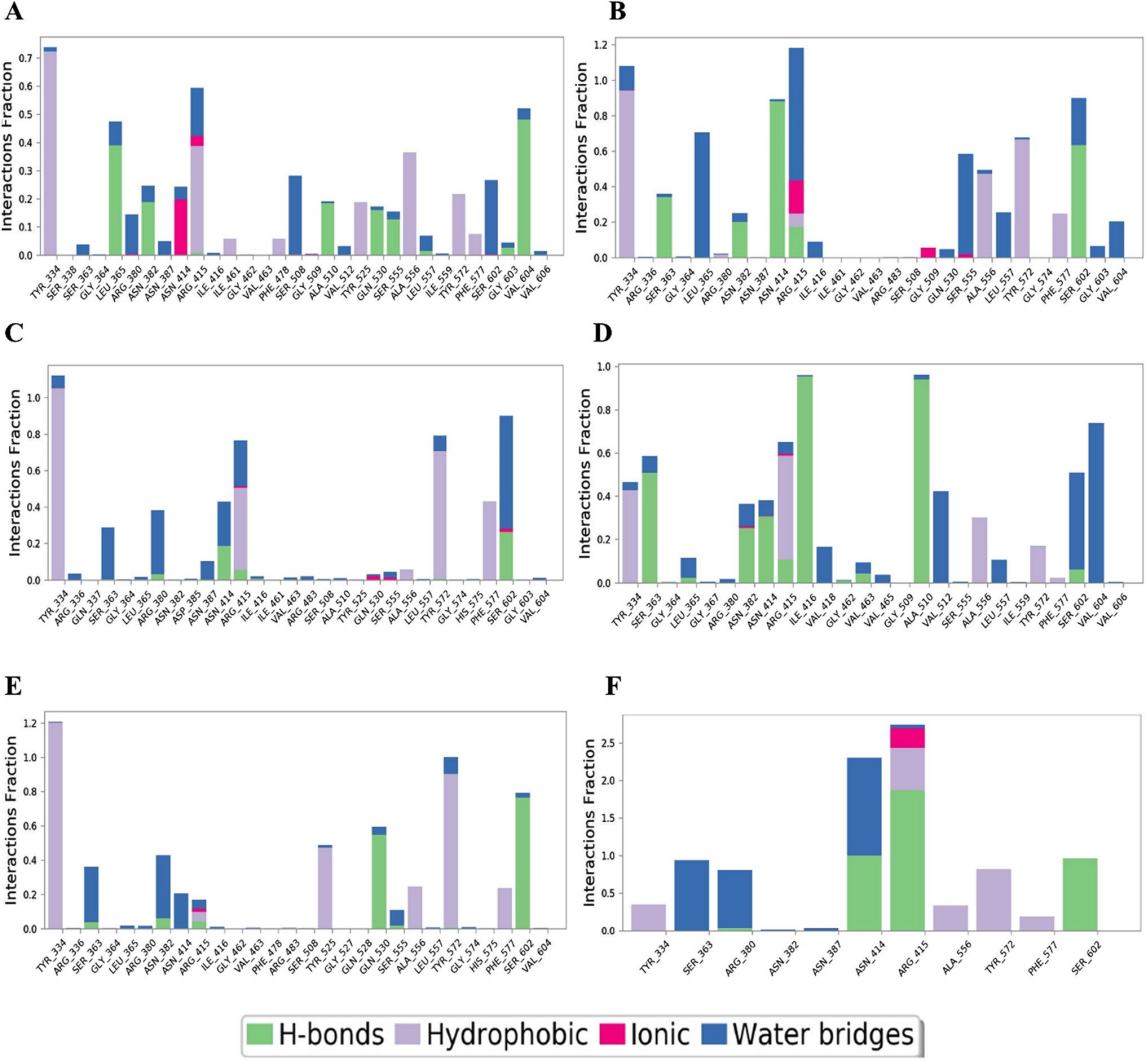
Contact histogram of—**a** Complex-1, **b** Complex-2, **c** Complex-3, **d** Complex-4, **e** Complex-5, and **f** Complex-6

**Fig. 6:**
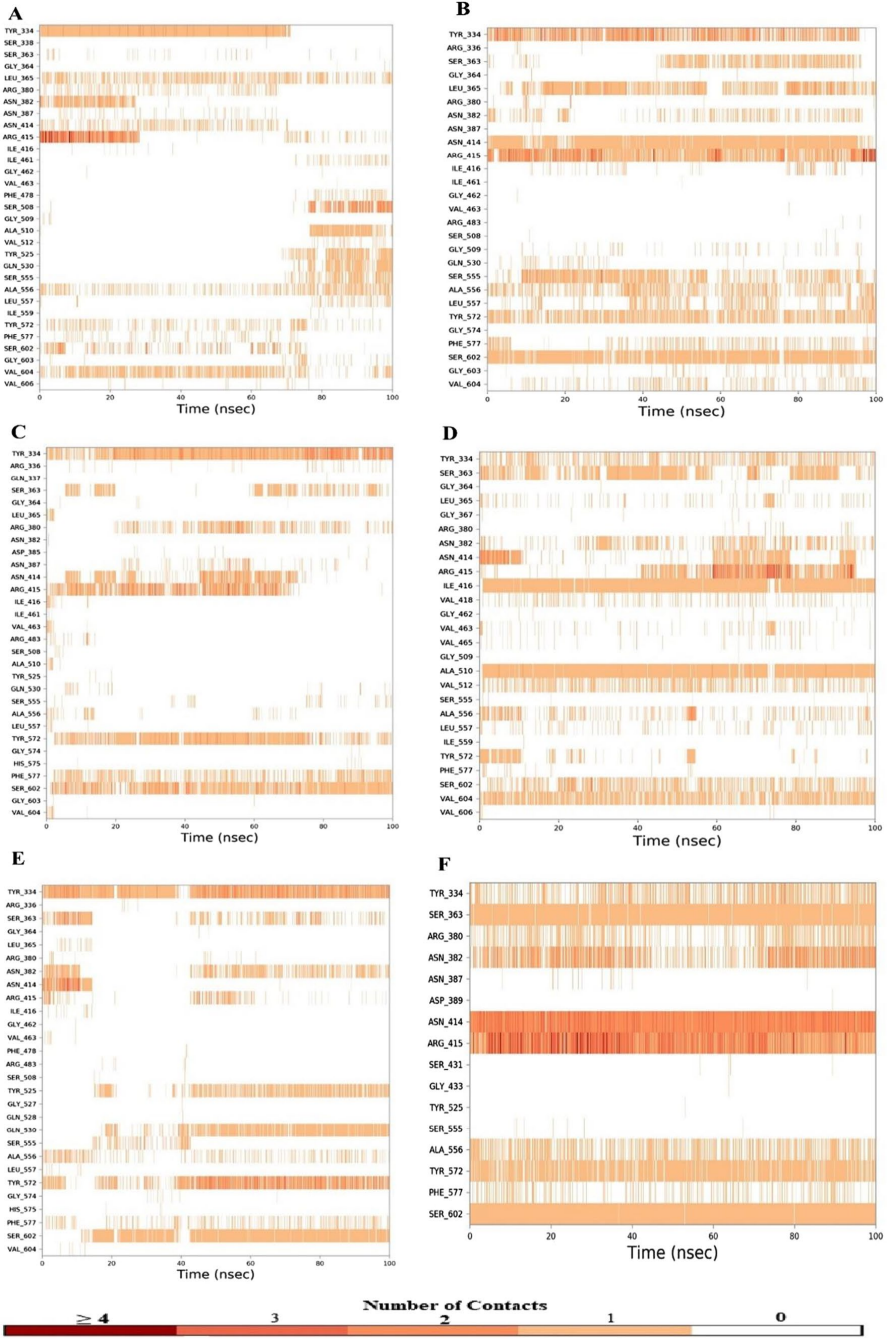
Contact timeline of—**a** Complex-1, **b** Complex-2, **c** Complex-3, **d** Complex-4, **e** Complex-5, and **f** Complex-6

The protein–ligand contact demonstrates the contact period between protein amino acids and ligand during the entire simulation. A score of 0.6 indicates that a contact was maintained for 60% of the simulation time, whereas a score greater than 1 indicates that amino acids residue of protein may form additional same subtype contacts with the ligand. In the complex-1, the residues VAL604, SER602, TYR572, ALA556, SER555, GLN530, TYR525, ALA510, SER508, ARG415, ASN414, ASN382, ARG380, LEU365, and TYR334 show interaction value of 0.52, 0.33, 0.22, 0.36, 0.15, 0.17, 0.19, 0.19, 0.28, 0.59, 0.15, 0.24, 0.25, 0.48, and 0.74, respectively. In complex-2, the residues VAL604, SER602, PHE577, TYR572, LEU557, ALA556, SER555, ARG415, ASN414, ASN382, LEU365, SER363, and TYR334 have interaction score of 0.20, 0.90, 0.25, 0.68, 0.26, 0.49, 0.59, 1.18, 0.89, 0.25, 0.71, 0.36, and 1.08, respectively. In the complex-3, the interaction values of 0.90, 0.43, 0.79, 0.77, 0.35, 0.38, 0.29 and 1.12 are shown by residues SER602, PHE557, TYR572, ARG415, ASN414, ARG380, SER363, and TYR334, respectively. In the complex-4, the residues VAL604, SER602, TYR572, ALA556, VAL512, ALA510, VAL418, ILE416, TYR334, ASN414, ARG415, ASN382, SER363 have interaction scores of 0.74, 0.51, 0.17, 0.30, 0.42, 0.96, 0.16, 0.96, 0.46, 0.38, 0.65, 0.36, and 0.58, respectively. In complex-5, residues SER602, PHE577, TYR572, ALA556, GLN530, TYR525, ARG415, ASN414, ASN382, SER363, and TYR334 have interaction values of 0.79, 0.24, 0.98, 0.25, 0.59, 0.49, 0.17, 0.21, 0.43, 0.36, and 1.20, respectively. In the complex-6, the interaction values of residues SER602, PHE557, TYR572, ALA556, ARG415, ASN414, ARG380, SER363, and TYR334 are 0.98, 0.20, 0.80, 0.30, 2.8, 2.3, 0.8, 0.98, and 0.3, respectively. Compound 46148111 of Complex-2 show better contact duration than the rest of the compounds. Contact duration of compound 46148111 was similar to that of 1VX (complex-6). The protein–ligand contact histogram and timeline of selected compounds (complex-1, complex-2, complex-3, complex-4, & complex-5) and co-crystallized ligand (complex-6) are shown in Figs. 5 and 6.

#### Hydrogen bond (H-bond) analysis

**Fig. 7:**
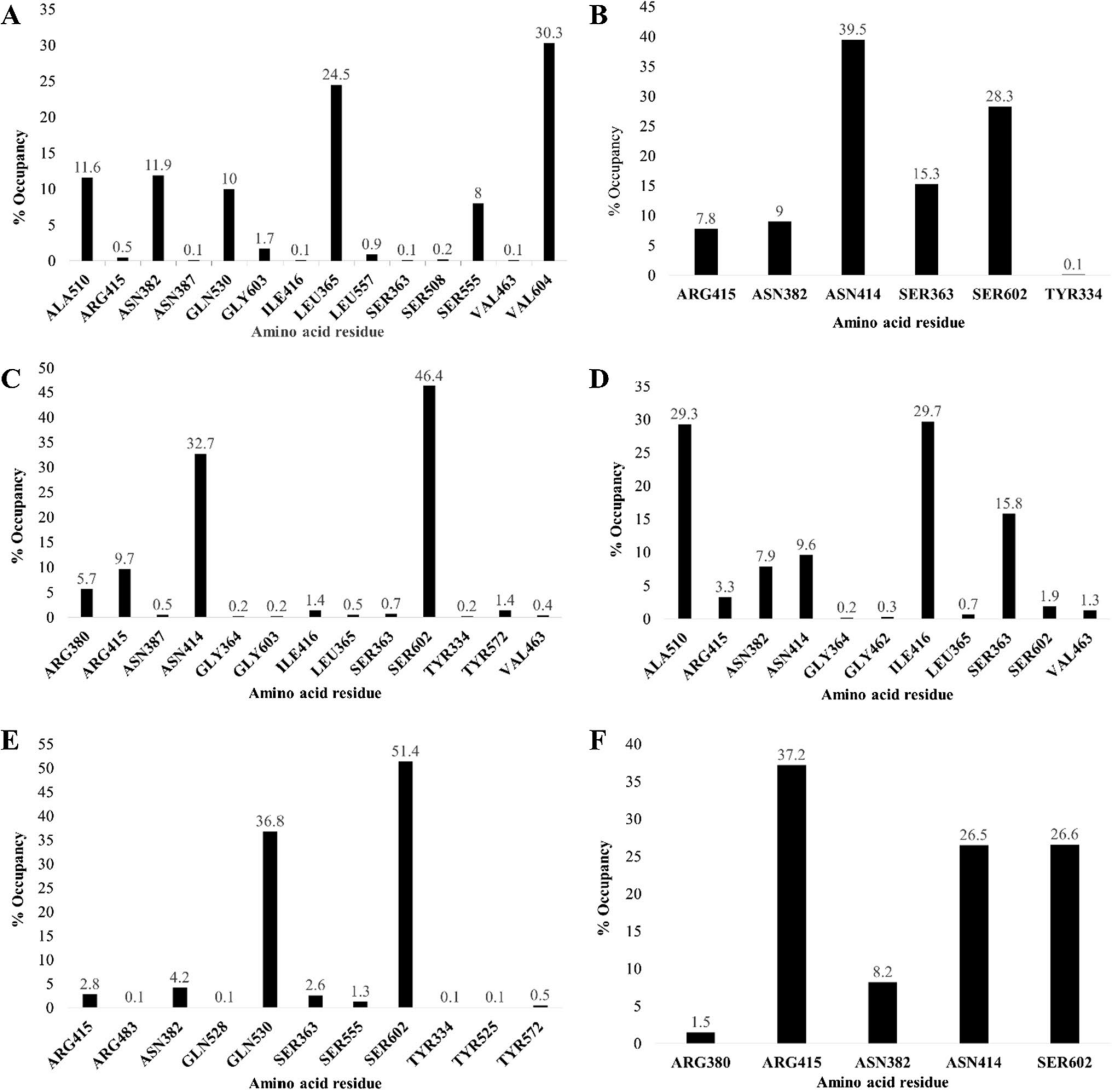
H-Bond histogram of—**a** Complex-1, **b** Complex-2, **c** Complex-3, **d** Complex-4, **e** Complex-5, and **f** Complex-6

For the protein–ligand complex stability, Hydrogen bond plays a critical role. H-Bond analysis echoes on how the ligands have interacted with the amino acids of the protein and the type of rearrangement that occurs in the binding site of protein upon ligand binding. The number of H-bonds was measured for each frame, i.e., 1000 frames for the entire duration of 100 ns simulation. In Complex-1, Ligand forms a total of 1585 H-bond during the entire simulation with the protein, where Amino acid residues ALA510, ASN382, GLN530, LEU365, SER555, and VAL604 show occupancy of 11.6%, 11.9%, 10%, 24.5%, 8%, and 30.3%, respectively. While amino acid residues ARG415, ASN387, GLY603, ILE416, LEU557, SER363, SER508, and VAL463 show 0.5%, 0.1%, 1.7%, 0.1%, 0.9%, 0.1%, 0.2%, and 0.1% occupancy, respectively. In complex-2, 2230 H-bond was formed between the ligand and protein, where Amino acid residues ARG415, ASN382, ASN414, SER363, SER602, and TRY334 show 7.8%, 9%, 39.5%, 15.3%, 28.3%, and 0.1% occupancy, respectively. In Complex-3, amino acid residues ARG380, ARG415, ASN387, ASN414, GLY364, GLY603, ILE416, LEU365, SER363, SER602, TYR334, TYR572, and VAL463 show occupancy of 5.7%, 9.7%, 0.5%, 32.7%, 0.2%, 0.2%, 1.4%, 0.5%, 0.7%, 46.4%, 0.2%, 1.4%, and 0.4%, respectively. In Complex-4, a total of 3210 H-bond was formed during the simulation, where amino acid residues ALA510, ARG415, ASN382, ASN414, GLY364, GLY462, ILE416, LEU365, SER363, SER602, and VAL463 show occupancy of 29.3%, 3.3%, 7.9%, 9.6%, 0.2%, 0.3%, 29.7%, 0.7%, 15.8%, 1.9%, and 1.3%, respectively. In Complex-5, 1487 H-bond was formed during the entire duration of the simulation, where amino acid residues ARG415, ARG483, ASN382, GLN528, GLN530, SER363, SER555, SER602, TYR334, TYR525, and TYR572 show 2.8%, 0.1%, 4.2%, 0.1%, 36.8%, 2.6%, 1.3%, 51.4%, 0.1%, 0.1%, and 0.5% occupancy, respectively. In Complex-6, 3754 H-bond was formed between the amino acid residue ARG380, ARG415, ASN382, ASN414, and SER602 with 1.5%, 37.2%, 8.2%, 26.5%, and 26.6% occupancy, respectively. The amino acid residue and % occupancy histogram are shown in Fig. 7.

#### Principal component analysis (PCA)

**Fig. 8:**
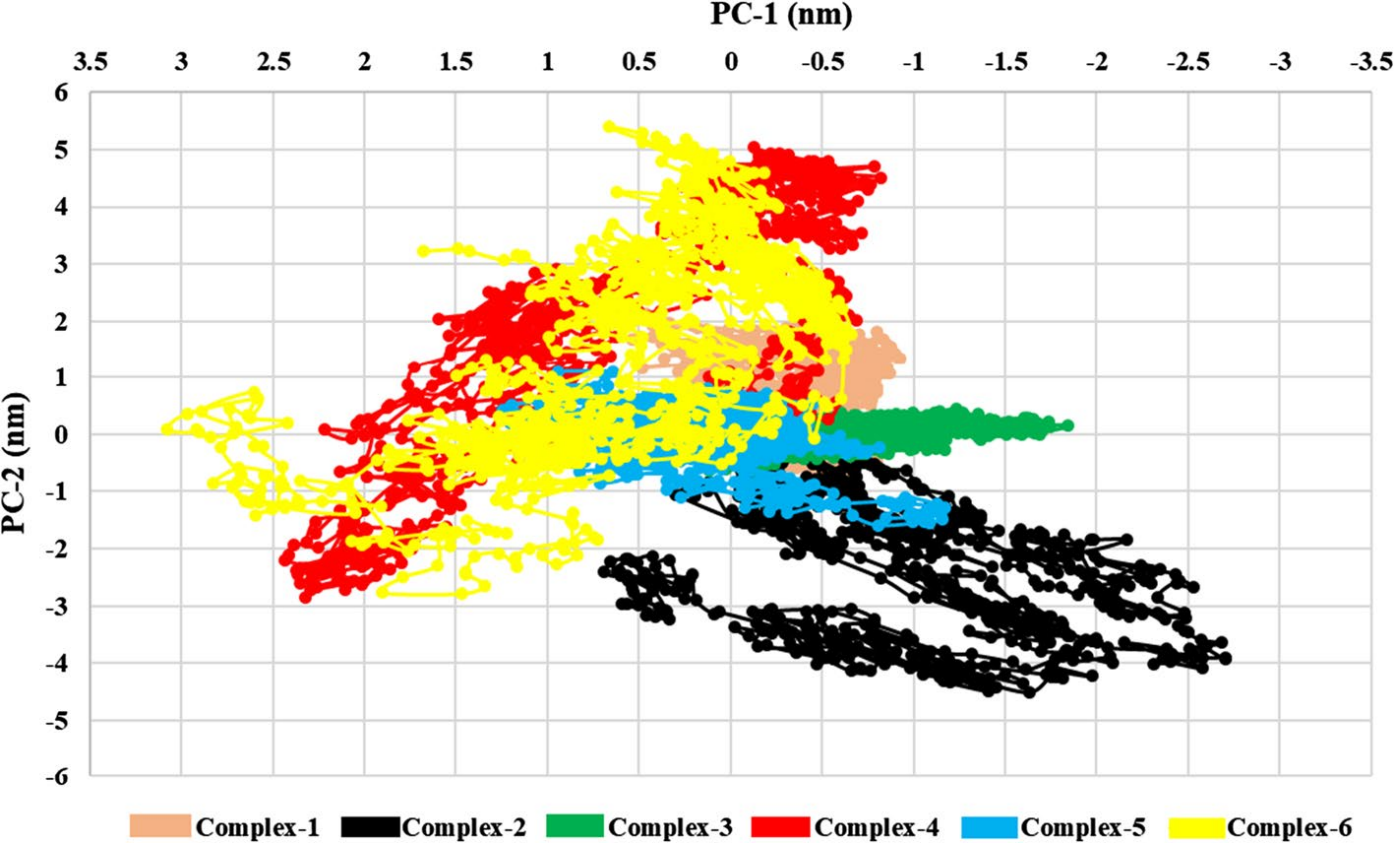
Scatter plot of PC1 vs PC2 of Complexes

Principal Component Analysis (PCA) is a highly effective technique for reducing complexity and extracting significant motions in the analysis of Molecular Dynamics (MD) simulations. In this approach, a matrix was constructed based on the trajectories, excluding rotational and translational movements. The essential dynamics protocol was then applied to compute the eigenvectors, eigenvalues, and their projections onto the first two Principal Components (PCs). By diagonalizing the matrix, the eigenvectors and eigenvalues were determined, with the eigenvalues representing the magnitude of the corresponding eigenvectors. The matrix of eigenvectors describes the multidimensional space, providing information about the displacement of atoms in the protein along each direction. Desmond trajectories obtained after MDS was converted using ProDy 2.0 for Visual comparative analysis using VMD’s Normal Mode Wizard plugin [43–46]. The PC1 and PC2 of the Complex-1 is in the range of 0.54 to − 0.93 nm & 2.13 to − 0.73 nm, Complex-2 was in the range of 0.69 to − 2.70 nm & 0.58 to − 4.49 nm, for Complex-3 it was in the range of 2.43 to − 0.82 nm & 0.44 to − 0.68 nm, Complex-4 is in the range of 2.43 to − 0.82 nm & 5.03 to − 2.85 nm, Complex-5 was in the range of 1.25 to − 1.16 nm & 1.08 to − 1.58 nm, and for the Co-crystalized ligand–protein complex (Complex-6) was in the range of 3.08 to − 0.66 nm & 5.39 to − 2.79 nm, respectively. The range of PC1 and PC2 for all the complexes were found to be less than that compared to that of Co-crystalized ligand complex (Complex-6), which points toward the better stability of selected ligands as compared to that of Co-crystalized Ligand. The scatter plot of PC1 vs PC2 is shown in Fig. 8**.**

#### Binding free energy (MM-GBSA dG bind)

**Fig. 9:**
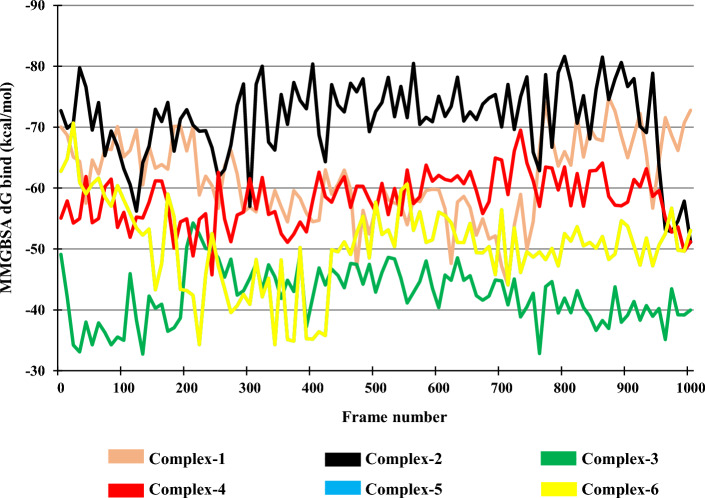
MM-GBSA of the Complexes

Post MDS, binding free energy of the selected complexes was calculated at every 10th frame for the duration of entire simulation, i.e., till the 1000th frame shown in Fig. 9**.** The lower the value of MM-GBSA dG bind, the more the protein–ligand complex stability. The average MM-GBSA dG bind scores for Complex-1, Complex-2, Complex-3, Complex-4, Complex-5, and Complex-6 were found to be − 61.59 kcal/mol, − 71.25 kcal/mol, − 42.45 kcal/mol, − 58.15 kcal/mol, − 50.30 kcal/mol, and − 63.79 kcal/mol, respectively. For Complex-1, Complex-2, Complex-3, Complex-4, Complex-5, and Complex-6, the MM-GBSA dG bind values lie in the range of − 74.88 to − 44.08 kcal/mol, − 81.64 to − 51.11 kcal/mol, − 54.29 to − 32.73 kcal/mol, − 69.53 to − 45.76 kcal/mol, − 70.68 to − 34.25 kcal/mol. and − 70.54 to − 57.26 kcal/mol, respectively. Minor fluctuation was observed in all the complexes with Complex-2 showing a better average MM-GBSA dG bind value than the co-crystalized Ligand (Complex-6).

#### Total energy

**Fig. 10:**
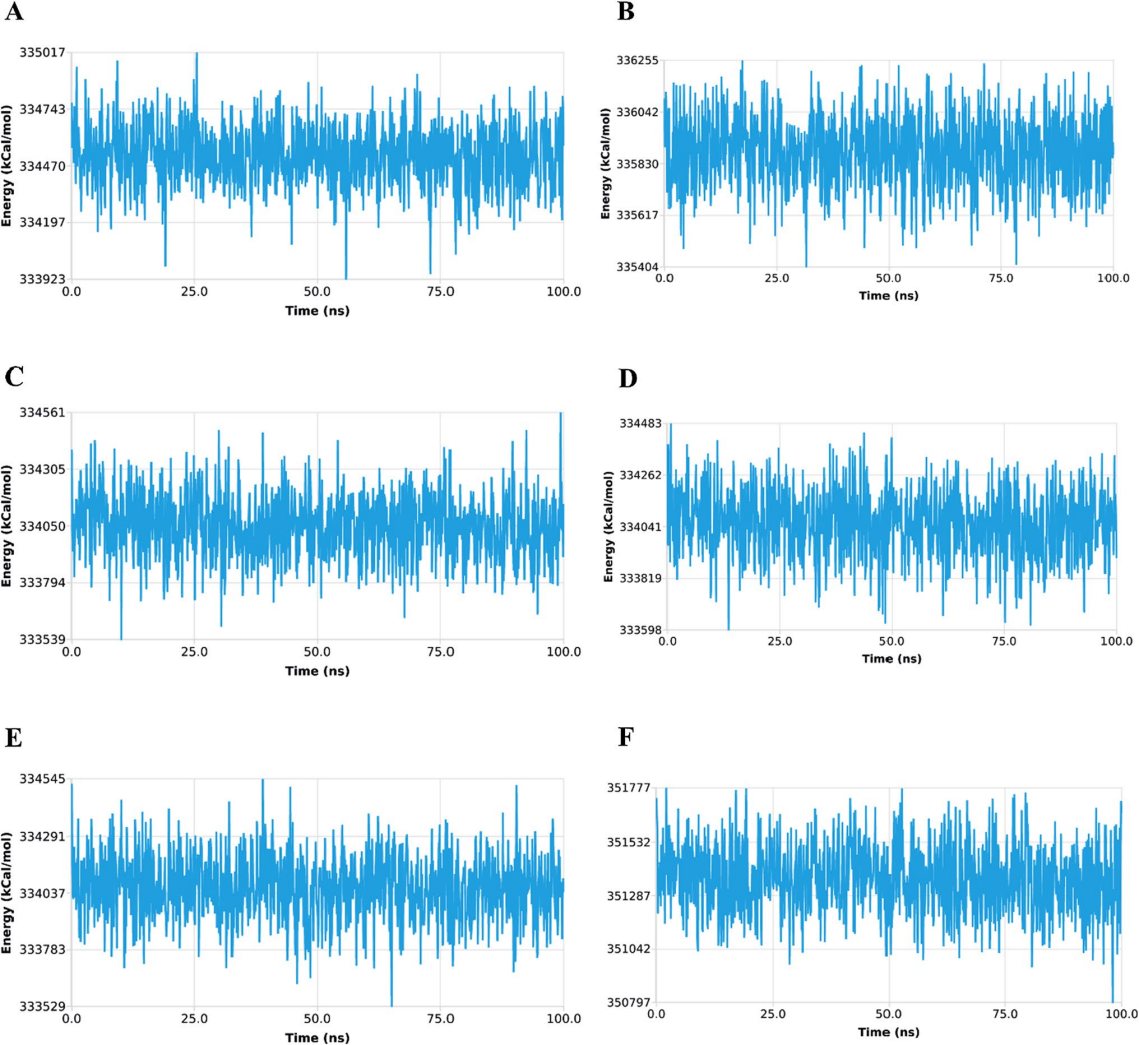
Total energy of—**a** Complex-1, **b** Complex-2, **c** Complex-3, **d** Complex-4, **e** Complex-5, and **f** Complex-6

The total energy (Coulomb, van der Waals, bond, angle, and dihedral) of the protein–ligand complex along with metals and ions, water, and hets groups during the molecular dynamic simulation is shown in Fig. 10**.** The total energy of Complex-1 lies in the range of 333923 to 335016 kcal/mol and the average value was 334518 kcal/mol. For Complex-2, the total energy lies in range of 335404 to 336255 kcal/mol, with average value of 335879 kcal/mol. The Complex-3 total energy lies in range of 333539 to 334560 kcal/mol, with average value of 334049 kcal/mol. For Complex-4, the total energy lies in range 333598 to 334483 kcal/mol, with average value of 334053 kcal/mol. For Complex-5, the total energy lies in range of 333528 to 334544 kcal/mol, with average value of 334060 kcal/mol. For Complex-6, the total energy lies in range of 350796 to 351776 kcal/mol, with average value of 335879 kcal/mol**.**

#### Solvent accessible surface area (SASA)

**Fig. 11:**
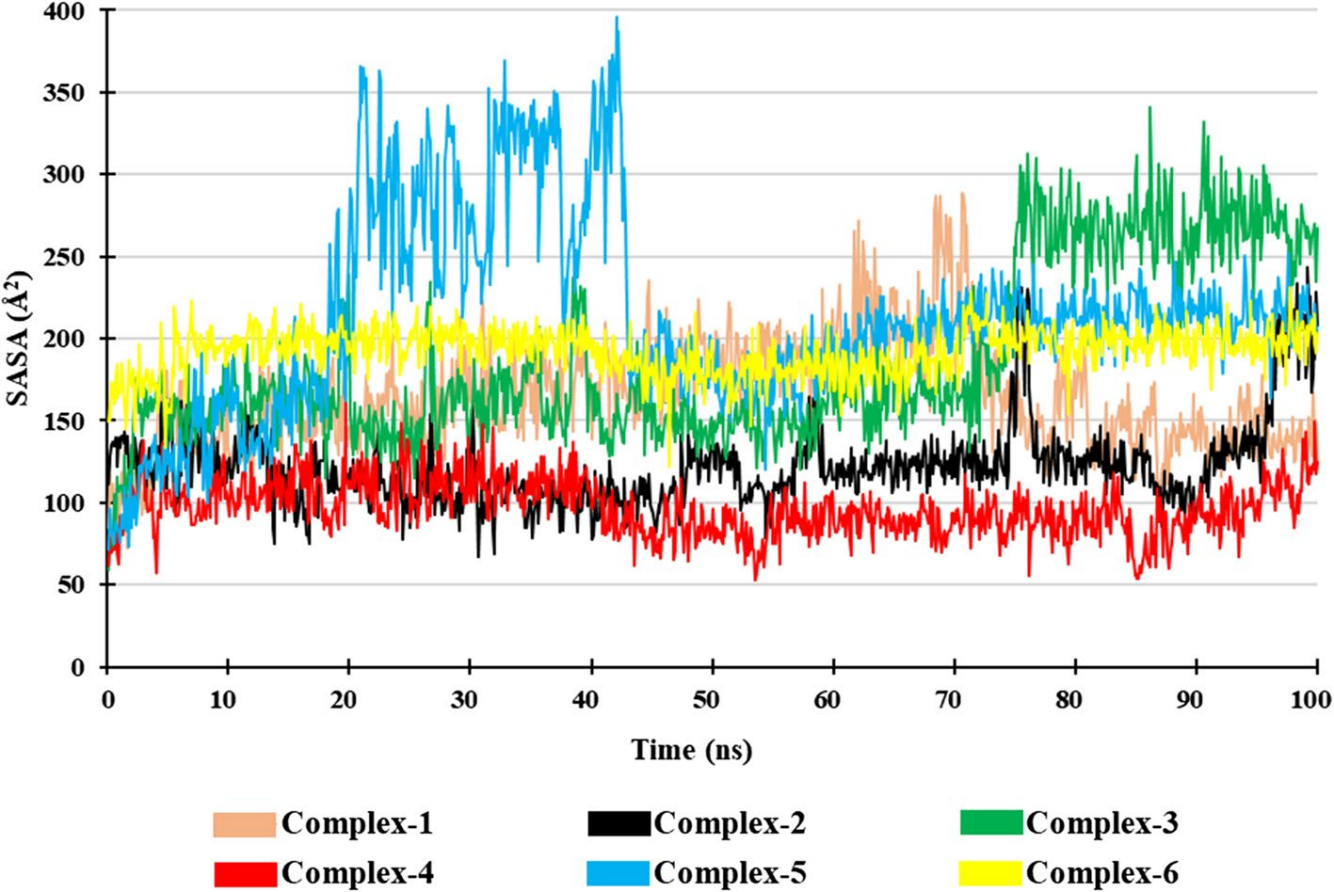
Solvent Accessible Surface Area (SASA) of the Complexes

Solvent accessible surface area (SASA) is defined as protein surface area, which are accessible to solvent. It helps in understanding the behavior of target protein when bounded with a ligand. SASA of the protein–ligand complex during the entire simulation was recorded and shown in Fig. 11. The average SASA values of Complex-1, Complex-2, Complex-3, Complex-4, Complex-5, and Complex-6 were around 164.25 Å^2^, 122.46 Å^2^, 186.31 Å^2^, 96.61 Å^2^, 212.34 Å^2^, and 192.16 Å^2^, respectively. SASA analysis shows that Complex-5 was stable in the initial phase, but it fluctuated during 18 to 43 ns, and it stabilized after 43 ns till the end of the simulation. All the other Complexes, i.e., Complex-1, Complex-2, Complex-3, and Complex-4, show a stable SASA value indicating a stable hydrophobic interaction between the ligand–receptor complexes, similar to that of co-crystalized ligand–protein complex (Complex-6).

#### Radius of gyration (Rg)

**Fig. 12:**
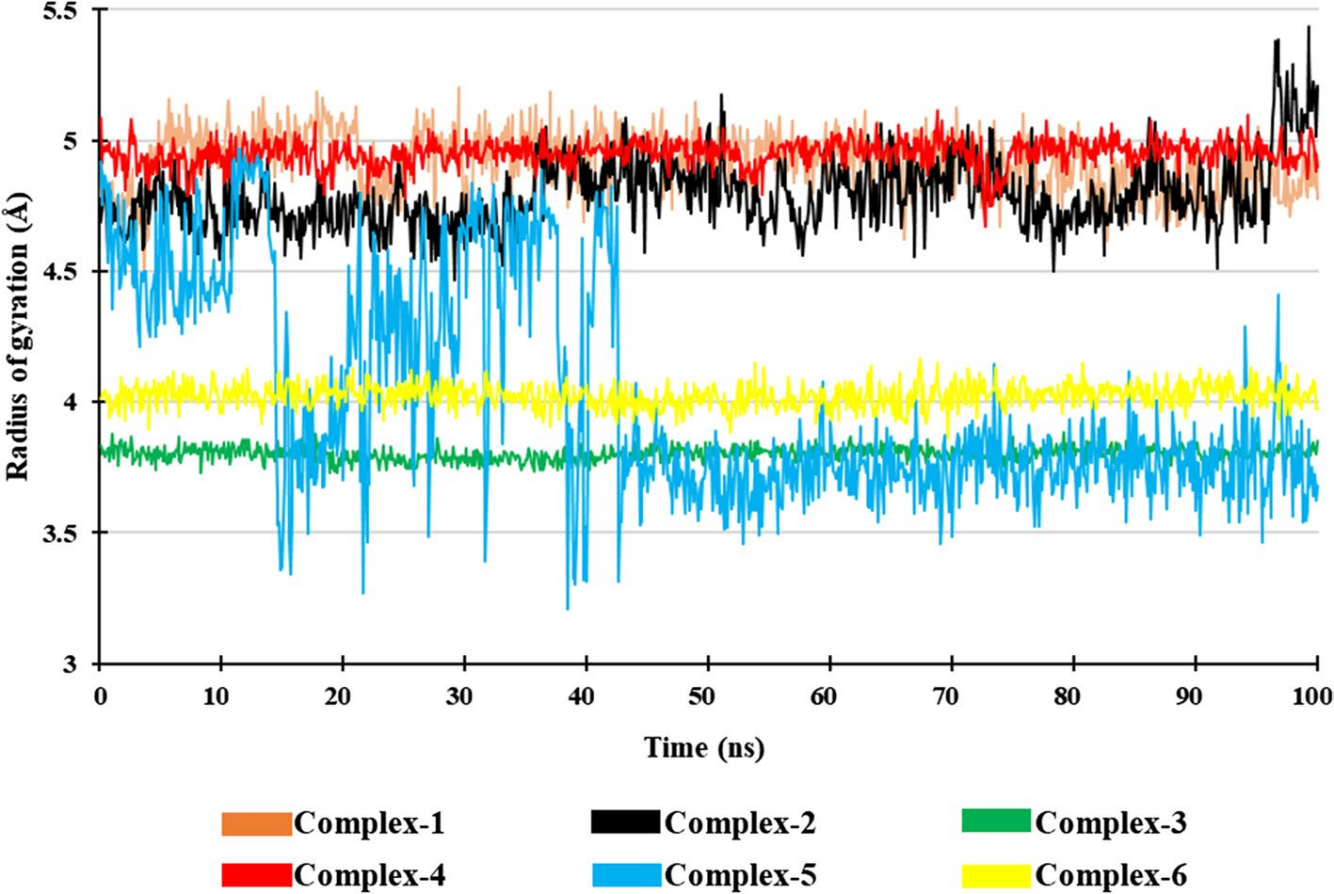
Radius of gyration of the Complexes

The radius of gyration helps in understanding the compactness of the protein–ligand complex. The constant and lower value of Rg indicates stable folding nature and compactness. The average Rg value of Complex-1 is 4.9 Å, Complex-2 is 4.79 Å, Complex-3 is 3.8 Å, Complex-4 is 4.95 Å, Complex-5 is 4.0 Å, and Complex-6 is 4.0 Å. The Rg values of the Complex-1, Complex-2, Complex-3, Complex-4, Complex-5, and Complex-6 were in the range of 4.5–5.2 Å, 4.5–5.4 Å, 3.7–3.9 Å, 4.7–5.1 Å, 3.2–4.96 Å, and 3.86–4.16 Å, respectively. In all the complexes except Complex-5, minor fluctuations were observed and they were stable during the entire simulation period. In Complex-5, from 14 to 42 ns slight fluctuation was observed but it becomes stable after 43 ns till the end of the simulation (Fig. 12).

## Conclusion

Any imbalance in the functioning of drug-metabolizing enzymes (phase-I and phase-II) can lead toward Drug-induced liver injury. The imbalance in the working of drug-metabolizing enzymes leads to the generation of ROS-induced oxidative stress leading to cell death. Sesamol is a phenolic compound isolated from *Sesamum indicum* L. seed oil. Sesamol has reported antioxidant and NRF2-modulating activity. In this study, computational technique was employed to identify Sesamol derivatives that can act as NRF2 activators. PDBs for KEAP1-NRF2 were downloaded from the RCSB databank. After careful evaluation, PDB id 4L7D was selected, having an RMSD of 0.8682 and 2.25 Å resolution. The co-crystalized ligand, 1VX was reported to disrupt the KEAP1-NRF2 protein–protein interaction by binding with KEAP1 protein at SER363, ARG (380 & 415), ASN (382 & 415), and TYR572 amino acids residue. 63345 compounds having Sesamol basic ring were downloaded from the PubChem database. XP-Ligand screening, MM-GBSA, ADMET, and Induced fit docking analysis were done to identify compounds interaction, docking score, binding free energy, bond retention, pharmacokinetic, and toxicity profile of the selected compound. Based on the above analysis, 5 compounds, 66867225, 46148111, 12444939, 123892179, and 94817569, were chosen for MDS studies. MDS studies give insight into the protein–ligand interaction mechanism. All the selected compounds interacted with TYR (334 & 572), SER (363 & 602), ASN (382 & 414), and ARG415 residue of the KEAP1 protein's active pocket, and they were stable. All the complexes also show good RMSF, PCA, Rg, SASA, RMSD, and total energy.

All the selected compounds show good binding free energy, pharmacokinetics, and toxicity profiles and follow Lipinski's rule of Five.

Based on the computational assay, the selected compounds 66867225, 46148111, 12,444,939, 123892179, and 94817569 can serve as an activator of NRF2. However, our result should be validated using a proper in-vivo/in-vitro experimental model of DILI.

## Abbreviations

ARG: Arginine
ASN: Asparagine
DILI: Drug-induced liver injury
DLG: Aspartic acid–Leucine–Glycine
ETGE: Glutamic acid–Threonine‐Glycine–Glutamic acid
GLN: Glutamine
HTVS: High-throughput virtual screening
IFD: Induced fit docking
ILE: Isoleucine
KEAP1: Kelch-like ECH-associated protein 1
LEU: Leucine
MDS: Molecular dynamic simulation
NRF2: Nuclear factor erythroid 2-related factor 2
RMSD: Root mean square deviation
RMSF: Root mean square fluctuation
PCA: Principal component analysis
SASA: Solvent accessible surface area
*R*_g_: Radius of gyration
ROS: Reactive oxygen species
SER: Serine
SP: Standard precision
TYR: Tyrosine
VAL: Valine
XP: Extra precision
